# Integrated analysis of randomized controlled trials evaluating bortezomib + lenalidomide + dexamethasone or bortezomib + thalidomide + dexamethasone induction in transplant-eligible newly diagnosed multiple myeloma

**DOI:** 10.3389/fonc.2023.1197340

**Published:** 2023-11-02

**Authors:** Laura Rosiñol, Benjamin Hebraud, Albert Oriol, Anne-Laurène Colin, Rafael Ríos Tamayo, Cyrille Hulin, María Jesús Blanchard, Denis Caillot, Anna Sureda, Miguel Teodoro Hernández, Bertrand Arnulf, Maria-Victoria Mateos, Margaret Macro, Jesús San-Miguel, Karim Belhadj, Juan José Lahuerta, M. Brigid Garelik, Joan Bladé, Philippe Moreau

**Affiliations:** ^1^ Department of Hematology, Hospital Clínic Institut d’investigacions Biomèdiques August Pi i Sunyer, Barcelona, Spain; ^2^ Hematology Department, Institut Universitaire du Cancer de Toulouse-Oncopole, Toulouse, France; ^3^ Institut Català d’Oncologia I Institut Josep Carreras, Hospital Germans Trias i Pujol, Badalona, Spain; ^4^ Service de Pharmacologie Médicale et Clinique, Centre Hospitalier et Universitaire de Toulouse, Toulouse, France; ^5^ Department of Hematology, Hospital Universitario Puerta de Hierro, Majadahonda, Spain; ^6^ Department of Hematology, Hôpital Haut-Lévêque, Bordeaux Pessac, France; ^7^ Department of Hematology, Hospital Ramón y Cajal, Madrid, Spain; ^8^ CHU Dijon, Hôpital du Bocage, Dijon, France; ^9^ Institut Català d’Oncologia-Hospitalet i Institut d’Investigació Biomèdica de Bellvitge (IDIBELL), Universitat de Barcelona, Barcelona, Spain; ^10^ Hematology Department, Hospital Universitario de Canarias, Santa Cruz de Tenerife, Spain; ^11^ Centre Hospitalier Universitaire, Hôpital St-Louis, Paris, France; ^12^ Hospital Universitario de Salamanca, Instituto de Investigación Biomédica de Salamanca, Salamanca, Spain; ^13^ Institut d’Hématologie de Basse Normandie, Centre Hospitalier et Universitaire de Caen, Caen, France; ^14^ Clínica Universidad de Navarra (CUN), Centro de Investigación Médica Aplicada (CIMA), Instituto de Investigación Sanitaria de Navarra (IDISNA), Centro de Investigación Biomédica en Red de Cáncer (CIBERONC), Pamplona, Spain; ^15^ Lymphoid Malignancies Unit, Centre Hospitalier et Universitaire Henri Mondor, Creteil, France; ^16^ Celgene, Bristol-Myers Squibb Company, Summit, NJ, United States; ^17^ Department of Hematology, University Hospital Hôtel-Dieu, Nantes, France

**Keywords:** multiple myeloma, bortezomib, lenalidomide, dexamethasone, thalidomide

## Abstract

**Objective:**

Providing the most efficacious frontline treatment for newly diagnosed multiple myeloma (NDMM) is critical for patient outcomes. No direct comparisons have been made between bortezomib + lenalidomide + dexamethasone (VRD) and bortezomib + thalidomide + dexamethasone (VTD) induction regimens in transplant-eligible NDMM.

**Methods:**

An integrated analysis was performed using patient data from four trials meeting prespecified eligibility criteria: two using VRD (PETHEMA GEM2012 and IFM 2009) and two using VTD (PETHEMA GEM2005 and IFM 2013-04).

**Results:**

The primary endpoint was met, with VRD demonstrating a noninferior rate of at least very good partial response (≥ VGPR) after induction *vs* VTD. GEM comparison demonstrated improvement in the ≥ VGPR rate after induction for VRD *vs* VTD (66.3% *vs* 51.2%; *P* = .00281) that increased after transplant (74.4% *vs* 53.5%). Undetectable minimal residual disease rates post induction (46.7% *vs* 34.9%) and post transplant (62.4% *vs* 47.3%) support the benefit of VRD *vs* VTD. Treatment-emergent adverse events leading to study and/or treatment discontinuation were less frequent with VRD (3%, GEM2012; 6%, IFM 2009) *vs* VTD (11%, IFM 2013-04).

**Conclusion:**

These results supported the benefit of VRD over VTD for induction in transplant-eligible patients with NDMM. The trials included are registered with ClinicalTrials.gov (NCT01916252, NCT01191060, NCT00461747, and NCT01971658).

## 1 Introduction

Multiple myeloma remains an incurable disease with relatively high mortality rates despite availability of multiple treatment options (1–3). Several treatment regimens are recommended for induction therapy in patients with transplant-eligible (TE) newly diagnosed multiple myeloma (NDMM) (4–6). Selection of the optimal frontline therapy is important, as 60% to 70% of patients receive fewer than three lines of therapy (7–10). Therefore, providing the most efficacious frontline therapy is critical to minimizing disease burden and optimizing survival outcomes (7–9, 11). Studies show that achieving at least very good partial response (≥ VGPR) during induction is associated with improved long-term outcomes (12–15). Other goals of induction therapy include achievement of rapid disease control and undetectable minimal residual disease (MRD) without negatively impacting stem cell harvest. Furthermore, low rates of toxicity would enable patients to complete induction, which helps optimize treatment responses.

National and international TE NDMM treatment guidelines, including European Society for Medical Oncology (ESMO), European Myeloma Network (EMN), and American Society of Clinical Oncology/Cancer Care Ontario (ASCO/CCO) guidelines, recommend triplet regimens such as bortezomib + lenalidomide + dexamethasone (VRD) and bortezomib + thalidomide + dexamethasone (VTD) (4–6). In addition, VRD and VTD are both currently being used as backbone therapy in modern quadruplet induction regimens with anti-CD38 monoclonal antibodies. However, in contrast to lenalidomide, the use of thalidomide is limited by peripheral neuropathy (16, 17). Bortezomib has also been associated with peripheral neuropathy, and the combination with thalidomide in VTD led to higher rates of this adverse event *vs* thalidomide + dexamethasone in phase 3 studies (18–20). The tolerability of bortezomib has improved with subcutaneous administration, which demonstrated noninferior efficacy and a lower incidence of peripheral neuropathy *vs* intravenous administration (21–23). Thus, VRD with subcutaneous bortezomib offers a treatment option with reduced rates of peripheral neuropathy. In phase 3 studies, VRD provided deep and durable responses during frontline therapy without limiting a patient’s ability to receive further therapy (24–28). Given these results, VRD has been integrated into clinical practice and is a preferred regimen for primary therapy for transplant candidates (4–6).

While both VRD and VTD are included as options for frontline therapy in international guidelines, no direct comparison of the safety and efficacy of VRD *vs* VTD has been done to date. In the absence of a randomized controlled trial (RCT), an integrated analysis can be performed using propensity score (PS)–based statistical methods (29, 30). This strategy minimizes the effects of observed baseline factors that could confound analysis to improve the comparison between different treatment cohorts. This method was previously used in a cross-trial comparison and regulatory submission to evaluate bortezomib ± dexamethasone in relapsed MM (31).

The goal of this integrated analysis was to compare VRD and VTD induction therapy in patients with TE NDMM. A literature search for phase 3 RCTs that met prespecified eligibility criteria identified two trials using VRD (PETHEMA GEM2012 and IFM 2009) and two trials using VTD (PETHEMA GEM2005 and IFM 2013-04). These four RCTs were included in the integrated analysis.

## 2 Methods

### 2.1 Study identification

A comprehensive review of published literature and ongoing clinical studies was performed to identify studies that met the following eligibility criteria: (1) study was a phase 3 RCT evaluating a full-dose VRD or VTD induction regimen (every 3 or 4 weeks) in patients with TE NDMM before autologous stem cell transplant (ASCT), (2) study had reached the primary endpoint for the purpose of the integrated analysis before data transfer, and (3) an agreement was in place for access to patient-level data adequate to conduct an integrated analysis by 31 December 2016. Search details are provided in the **Supplement**.

### 2.2 Endpoints

The primary endpoint of the integrated analysis was noninferiority of the post induction ≥ VGPR rate based on International Myeloma Working Group criteria. Secondary endpoints were safety, post ASCT ≥ VGPR rate, and ≥ VGPR rate over time during induction. Exploratory endpoints were progression-free survival (PFS), overall survival (OS), and undetectable MRD.

### 2.3 Statistical analysis

Statistical methods were based on the PS, which is a conditional probability of being treated given observed covariates that could be used to balance the covariates in two treatment cohorts and reduce bias (30). A logistic regression model in which treatment group was regressed based on 11 baseline variables (age, sex, height, weight, performance status, International Staging System disease stage, hemoglobin level, creatinine clearance, albumin level, β2-microglobulin level, and lactate dehydrogenase level) was used to estimate PS (32). Patients with missing baseline values for any of these variables were excluded. Patients were stratified into five equal-sized groups using the quintiles of the estimated PS. Additional details on the PS model and noninferiority hypothesis are provided in the **Supplement**.

To demonstrate noninferiority of VRD *vs* VTD, a margin of noninferiority was prespecified using a two-sided 95% confidence interval. For the primary endpoint, post-initial treatment response rate of ≥ VGPR, the noninferiority margin (11.3%) was selected using historical data; a margin of 10% did not represent a substantial difference in treatment effect and was within normal variance between two treatment regimens in similar patient populations.

For PFS and OS, Kaplan-Meier methodology was used for the descriptive statistics. The stratified log-rank test, with the stratum based on the quintiles of the PS, was used to assess statistical significance of treatment effects. The stratified Cox proportional hazards model was used to estimate the hazard ratio and 95% CI for VRD *vs* VTD.

In GEM2005, MRD was assessed using four-color multiparameter flow cytometry with undetectable MRD defined as < 20 clonal plasma cells after measuring ≥ 200,000 nucleated cells at a sensitivity level of 10^−4^. GEM2012 assessed MRD at sensitivity levels of 10^−4^ and 10^−6^ using next-generation flow following EuroFlow protocols per International Myeloma Working Group, using an optimized eight-color, two-tube antibody panel (33).

### 2.4 Safety analysis

The safety analysis was primarily based on the induction phase of treatment, and the safety population included all randomized patients who received at least one dose of study drug. Adverse events were coded using the Medical Dictionary for Regulatory Activities version 15.1 and summarized by system organ class and preferred terms. Adverse events were graded using the National Cancer Institute Common Terminology Criteria for Adverse Events versions 4.03 (PETHEMA GEM2012) and 4.0 (IFM studies). Due to limited access to some data from the PETHEMA GEM2005 clinical trial database, safety data from that study are not included here. Treatment-emergent peripheral neuropathy was summarized using the grouped term (see **Supplement**).

## 3 Results

### 3.1 Study identification

**Figure 1:**
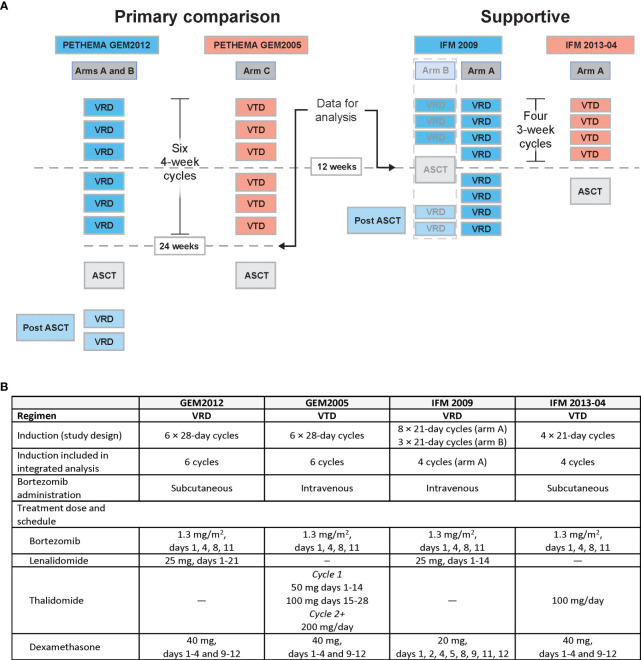
Design of studies meeting eligibility criteria. **(A)** Graphical overview and **(B)** tabular summary of GEM and IFM study designs. ASCT, autologous stem cell transplant; MRD, minimal residual disease; VRD, bortezomib, lenalidomide, and dexamethasone; VTD, bortezomib, thalidomide, and dexamethasone.

Sixteen studies of prospective phase 3 RCTs evaluating VRD or VTD induction (every 3 or 4 weeks) in patients with TE NDMM before ASCT were identified. A brief overview of the study details and the criteria not met for inclusion for 12 trials are provided in **Table S1**. Four studies met the eligibility criteria and were included: PETHEMA GEM2012 and IFM 2009 for VRD and PETHEMA GEM2005 and IFM 2013-04 for VTD (**Figure 1A**) (16, 18, 24, 25).

Similarities and differences in the induction phase (length, number, dose, schedule, and route of bortezomib administration) are summarized for the included studies in **Figure 1B**. For example, GEM studies used 28-day cycles, whereas IFM studies used 21-day cycles. Additionally, although the bortezomib dose was the same for all four studies, bortezomib was administered subcutaneously in GEM2012 and IFM2013-04 *vs* intravenously in GEM2005 and IFM 2009. Lenalidomide was given on days 1 to 21 of the 28-day cycle in GEM2012 *vs* days 1 to 14 of the 21-day cycle in IFM 2009. In GEM2005, the thalidomide dose was escalated from 50 mg/day to 100 mg/day in cycle 1 and 200 mg/day thereafter; whereas, in IFM 2013-04, it was 100 mg/day throughout induction. Furthermore, differences between the induction regimens affected how data were included in the integrated analysis. For example, GEM2012 (VRD) and GEM2005 (VTD) were considered the main studies for the efficacy analysis due to their symmetrical induction regimens (six 4-week cycles of induction followed by ASCT). IFM 2009 (VRD) and IFM 2013-04 (VTD) were considered supportive studies due to differences in design, meaning that these studies were included in the efficacy analysis to support the main efficacy comparisons made for the GEM studies. While both used 3-week cycles, IFM 2009 arm A used eight cycles, arm B used three cycles followed by ASCT and two cycles of consolidation, and IFM 2013-04 used four cycles followed by ASCT. Based on these differences in the IFM studies, only IFM 2009 arm A was used to compare with IFM 2013-04. Only data through four cycles of induction were included. This comparison of studies was possible because of the research agreement granting patient-level data access for each study.

Eligibility criteria were similar between the included studies. The four studies were all conducted in patients < 65 years of age with newly diagnosed, untreated MM with measurable M-protein concentrations (16, 18, 24, 25). Patients in these studies all had an Eastern Cooperative Oncology Group (ECOG) performance status of ≤ 3. GEM2012, GEM2005, and IFM2009 studies included patients with platelet counts of ≥ 50 to 100 × 10^9^/L and neutrophil counts of ≥ 1 × 10^9^/L. Additionally, GEM2012, IFM2009, and IFM2013-04 all excluded patients with grade ≥ 2 peripheral neuropathy.

### 3.2 PS-stratified population and baseline characteristics

In the GEM studies, the intent-to-treat populations included 458 patients who received VRD (GEM2012) and 130 patients who received VTD (GEM2005). Due to missing data for at least one baseline variable, 51 and 1 patients were excluded from the respective PS-stratified cohorts, leaving 407 (GEM2012) and 129 (GEM2005) patients in the integrated analysis. Similarly, intent-to-treat populations of the IFM studies had 19 and 15 patients, respectively, excluded from the respective PS-stratified cohorts due to missing data for at least one baseline variable. Thus, as 350 patients received VRD in IFM 2009 arm A and 169 received VTD in IFM 2013-14, 331 (IFM 2009) and 154 (IFM 2013-04) patients remained in the integrated analysis. The distributions of the overall PS of the VRD *vs* VTD cohorts for both GEM and IFM studies were similar (**Table S2**). Baseline characteristics were similar between the VRD and VTD PS-stratified cohorts (**Table 1**) and the respective overall intent-to-treat populations (**Table S3**).

**Table 1:** Baseline patient and disease characteristics.

	VRD GEM2012 n = 407	VTD GEM2005 n = 129	VRD IFM 2009 n = 331	VTD IFM 2013-04 n = 154
Median age, years	57	57	59	59
Range	31-65	33-65	28-65	34-65
Male, n (%)	211 (51.8)	75 (58.1)	196 (59.2)	93 (60.4)
ECOG PS, n (%)				
0	179 (44.0)	63 (48.8)	153 (46.2)	71 (46.1)
1	161 (39.6)	50 (38.8)	141 (42.6)	66 (42.9)
≥ 2	67 (16.5)	16 (12.4)	37 (11.2)	17 (11.0)
ISS stage, n (%)				
I	164 (40.3)	44 (34.1)	114 (34.4)	32 (20.8)
II	146 (35.9)	57 (44.2)	155 (46.8)	89 (57.8)
III	97 (23.8)	28 (21.7)	62 (18.7)	33 (21.4)
Cytogenetic risk, n (%)				
Standard	184 (45.2)	58 (45.0)	204 (61.6)	113 (73.4)
High^†^	78 (19.2)	23 (17.8)	35 (10.6)	28 (18.2)
Missing^‡^	145 (35.6)	48 (37.2)	92 (27.8)	13 (8.4)
LDH, n (%)				
Elevated	61 (15.0)	17 (13.2)	144 (43.5)	57 (37.0)
Not elevated	346 (85.0)	112 (86.8)	187 (56.5)	97 (63.0)
CrCl group, n (%)				
< 50 mL/min	34 (8.4)	8 (6.2)	8 (2.4)	9 (5.8)
≥ 50 mL/min	373 (91.6)	121 (93.8)	323 (97.6)	145 (94.2)

^†^ High risk = presence of del(17p), t(4;14), or t(14;16) in the GEM2012, GEM2005, and IFM 2009 studies and presence of del(17p) and/or t(4;14) in the IFM 2013-04 study.

^‡^ Missing = FISH failure in IFM studies and includes “other” in GEM studies.

CrCl, creatinine clearance; ECOG PS, Eastern Cooperative Oncology Group performance status; FISH, fluorescence in situ hybridization; ISS, International Staging System; LDH, lactate dehydrogenase; VRD, bortezomib, lenalidomide, and dexamethasone; VTD, bortezomib, thalidomide, and dexamethasone.

### 3.3 Response

**Figure 2:**
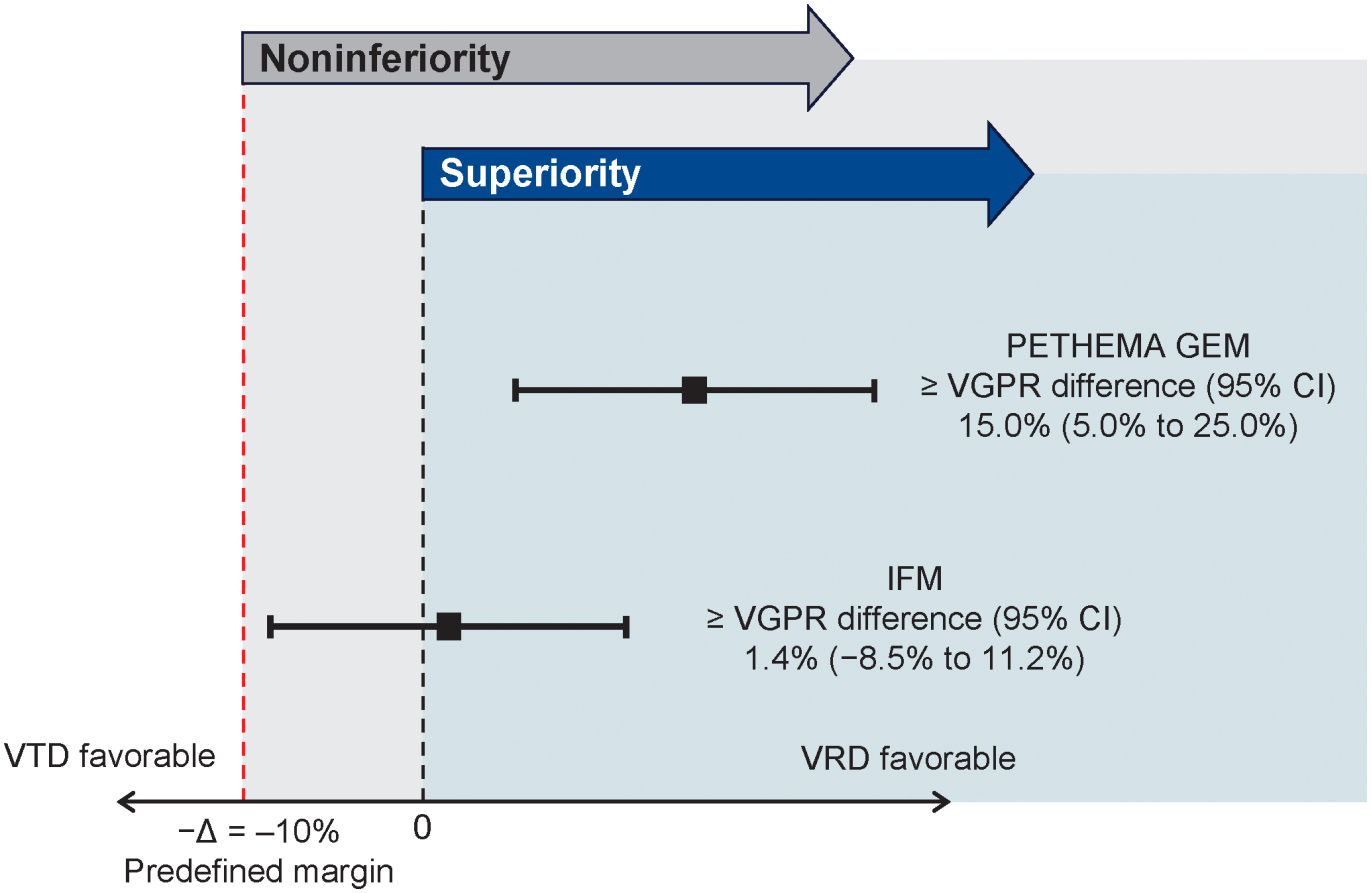
Noninferiority of VRD *vs* VTD. VGPR, very good partial response; VRD, bortezomib, lenalidomide, and dexamethasone; VTD, bortezomib, thalidomide, and dexamethasone.

The primary endpoint of noninferiority for VRD *vs* VTD was met in both the GEM and IFM analyses. In the primary comparison (GEM2012 *vs* GEM2005), the ≥ VGPR rate after six cycles of induction with VRD *vs* VTD was 66.3% *vs* 51.2%, respectively (odds ratio, 1.87 [95% CI, 1.23-2.83]; *P* = .00281) (**Table 2**), which was similar in most subgroups (**Figure S1**). In the GEM comparison, the ≥ VGPR difference was 15.0% (95% CI, 5.0%-25.0%) (**Figure 2**). Since the 95% CI was entirely above zero, superiority of VRD over VTD was concluded. In the IFM studies, the ≥ VGPR rate by four cycles (12 weeks) was noninferior with VRD (57.1%) *vs* VTD (56.5%) in the PS-stratified population, with similar results in patient subgroups (**Figure S2**).

**Table 2:** Response.

	**VRD** **GEM2012** **n = 407**	**VTD** **GEM 2005** **n = 129**	**Odds Ratio (95% CI)**
≥ VGPR, n (%)			
Post induction	270 (66.3)	66 (51.2)	1.87 (1.23-2.83)^†^
Post ASCT	303 (74.4)	69 (53.5)	2.52 (1.64-3.87)
Undetectable MRD (10^-4^ threshold), n (%)			
Post induction	190 (46.7)	45 (34.9)	1.39 (0.87-2.22)^‡^
Post ASCT	254 (62.4)	61 (47.3)	1.70 (0.94-3.05)^‡^
≥ VGPR and undetectable MRD (10^-4^ threshold), n (%)			
Post induction	171 (42.0)	34 (26.4)	1.49 (0.81-2.76)^‡^
Post ASCT	240 (59.0)	46 (35.7)	1.98 (0.93-4.20)^‡^
	IFM 2009 n = 331	IFM 2013-04 n = 154	
≥ VGPR post induction, n (%)	189 (57.1)	87 (56.5)	1.06 (0.71-1.59)

^†^ P = .00281.

^‡^ Dichotomized response; patients without any MRD assessment data were not included in the detectable category.

ASCT, autologous stem cell transplant; MRD, minimal residual disease; NA, not assessed; VGPR, very good partial response; VRD, bortezomib, lenalidomide, and dexamethasone; VTD, bortezomib, thalidomide, and dexamethasone.

**Figure 3:**
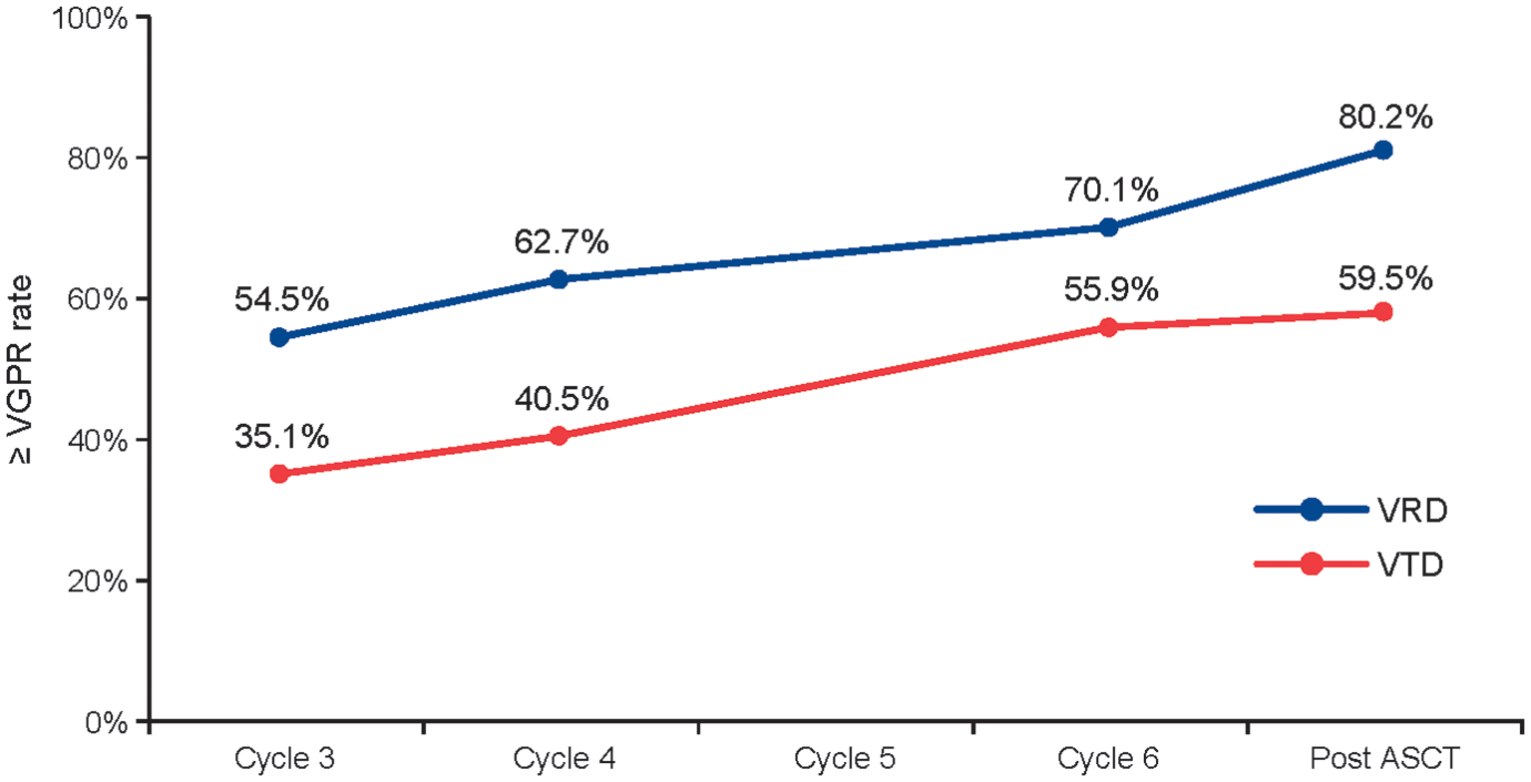
≥ VGPR rate throughout induction and post ASCT in the GEM studies. Data are based on the 378 patients taking VRD and 111 patients taking VTD who started cycle 6 in the GEM2012 and GEM2005 studies, respectively. ASCT, autologous stem cell transplant; PS, propensity score; VGPR, very good partial response; VRD, bortezomib, lenalidomide, and dexamethasone; VTD, bortezomib, thalidomide, and dexamethasone.

In the GEM studies, the ≥ VGPR rate increased over time with both regimens during induction. Among patients in the PS-stratified cohort who initiated cycle six, the ≥ VGPR rate improved from 54.5% at cycle three to 70.1% at cycle six with VRD (n = 378) and from 35.1% to 55.9% with VTD (n = 111) (**Figure 3**).

In the overall PS-stratified cohort in the GEM studies, the improvement in ≥ VGPR rate seen for VRD *vs* VTD post induction was maintained after ASCT (74.4% *vs* 53.5%). Of note, the ≥ VGPR rate improved more for VRD than for VTD from post induction to post ASCT (8% *vs* 2%). The undetectable MRD rates (10^–4^ threshold) post induction (46.7% *vs* 34.9%) and post ASCT (62.4% *vs* 47.3%) supported the benefit with VRD *vs* VTD. Data with a threshold of 10^–6^ were available for GEM2012, showing an undetectable MRD rate of 28.5% post induction and 41.8% post ASCT. Similar comparisons could not be performed with the IFM studies, as response over time and MRD were not assessed in IFM 2013-04.

### 3.4 Survival

Due to differences in median follow-up times and numbers of patients who experienced progression or died between studies, the 2-year event-free rate is a better comparison for the cohorts than median PFS or OS. In the GEM studies, the 2-year PFS rates (ie, those patients who had not experienced progression or died) were 82% and 69%, and the 2-year OS rates (ie, patients who had not died) were 90% and 87% with VRD and VTD, respectively.

In the IFM studies, the 2-year PFS rates were 67% and 71%, and the 2-year OS rates were 93% and 93% with VRD and VTD, respectively. However, differences in the number of cycles of induction received, inclusion of ASCT following induction, and treatment received post ASCT may limit interpretation of these data.

### 3.5 Safety

A summary of treatment-emergent adverse events (TEAEs) is provided in **Table 3**. The most common grade 3/4 (TEAE) in the GEM2012 VRD study was neutropenia (13%), and in the IFM studies, lymphopenia (49% with VRD and 22% with VTD; **Table 4**). In GEM2012, the rate of peripheral neuropathy was 21% for grade ≥ 2 events and 5% for grade 3/4 events. In the IFM studies, in which intravenous bortezomib was used in VRD and subcutaneous bortezomib was used in VTD, rates of grade ≥ 2 peripheral neuropathy events (30% *vs* 27% with VRD *vs* VTD, respectively) and grade 3/4 events (6% *vs* 8%, respectively) were similar.

**Table 3:** Summary of TEAEs^†^.

	VRD GEM2012 n = 458	
Patients with ≥ 1:		
TEAE	402 (87.8)	
Treatment-emergent SAE	147 (32.1)	
Most common any-grade TEAEs (≥ 10% of patients in any cohort)		
Peripheral neuropathy	160 (34.9)	
Neutropenia	146 (31.9)	
Infection	129 (28.2)	
Thrombocytopenia	116 (25.3)	
Skin toxicity	91 (19.9)	
Anemia	69 (15.1)	
Diarrhea	59 (12.9)	
Asthenia	56 (12.2)	
Constipation	55 (12.0)	
Neuralgia	25 (5.5)	
Pneumonia	24 (5.2)	
Pyrexia	21 (4.6)	
Peripheral edema	15 (3.3)	
Dizziness	7 (1.5)	
Paresthesia	5 (1.1)	
Nasopharyngitis	4 (0.9)	
Edema	2 (0.4)	
	VRD IFM 2009 n = 356	VTD IFM 2013-04 n = 169
Patients with ≥ 1:		
TEAE	354 (99.4)	164 (97.0)
Treatment-emergent SAE	98 (27.5)	54 (32.0)
Most common any-grade TEAEs (≥ 10 of patients in any cohort)		
Lymphopenia	178 (50.0)	41 (24.3)
Neutropenia	158 (44.4)	21 (12.4)
Peripheral sensory neuropathy	157 (44.1)	13 (7.7)
Fatigue	129 (36.2)	3 (1.8)
Constipation	126 (35.4)	55 (32.5)
Leukopenia	125 (35.1)	6 (3.6)
Nausea	106 (29.8)	38 (22.5)
Diarrhea	101 (28.4)	8 (4.7)
Peripheral edema	81 (22.8)	31 (18.3)
Insomnia	81 (22.8)	7 (4.1)
Thrombocytopenia	70 (19.7)	11 (6.5)
Rash	69 (19.4)	14 (8.3)
Paresthesia	67 (18.8)	26 (15.4)
Pyrexia	65 (18.3)	11 (6.5)
Anemia	60 (16.9)	16 (9.5)
Back pain	59 (16.6)	17 (10.1)
Vomiting	54 (15.2)	12 (7.1)
Headache	50 (14.0)	9 (5.3)
Muscle spasms	38 (10.7)	3 (1.8)
Neuropathy peripheral	13 (3.7)	51 (30.2)
Asthenia	3 (0.8)	36 (21.3)

^†^Safety was assessed using NCI CTCAE v4.03 (PETHEMA GEM2012) and v4.0 (IFM 2009 and IFM 2013-04). Due to limited access to some data from the PETHEMA GEM2005 clinical trial database, safety data from that study is not included here.

NCI CTCAE, National Cancer Institute Common Terminology Criteria for Adverse Events; SAE, serious adverse event; TEAE, treatment-emergent adverse event; VRD, bortezomib, lenalidomide, and dexamethasone; VTD, bortezomib, thalidomide, and dexamethasone.

**Table 4:** Grade 3/4 TEAEs in ≥5% of patients^†^.

	VRD GEM2012 n = 458	
Grade 3/4 TEAEs, n (%)	183 (40.0)	
Neutropenia	59 (12.9)	
Thrombocytopenia	29 (6.3)	
Infections and infestations	45 (9.8)	
Peripheral neuropathy^‡^	21 (4.6)	
	VRD IFM 2009 n = 356	VTD IFM 2013-04 n = 169
Grade 3/4 TEAEs, n (%)	296 (83.1)	101 (59.8)
Lymphopenia	176 (49.4)	38 (22.5)
Neutropenia	155 (43.5)	19 (11.2)
Leukopenia	124 (34.8)	6 (3.6)
Thrombocytopenia	65 (18.3)	6 (3.6)
Anemia	26 (7.3)	6 (3.6)
Peripheral neuropathy^‡^	21 (5.9)	13 (7.7)

^†^ Safety was assessed using NCI CTCAE v4.03 (PETHEMA GEM2012) and v4.0 (IFM 2009 and IFM 2013-04). Peripheral neuropathy of any percentage is included. Due to limited access to some data from the PETHEMA GEM2005 clinical trial database, safety data from that study is not included here.

^‡^ Grouped term used to capture events related to peripheral neuropathy.

NCI CTCAE, National Cancer Institute Common Terminology Criteria for Adverse Events; TEAE, treatment-emergent adverse event; VRD, bortezomib, lenalidomide, and dexamethasone; VTD, bortezomib, thalidomide, and dexamethasone.

### 3.6 Dose reductions and discontinuations

In GEM2012, 22% of patients had at least one TEAE leading to dose reduction, most commonly peripheral neuropathy (17%). At least one TEAE leading to study or treatment discontinuation occurred in 3% of patients with VRD and were most frequently due to infection (1%), septic shock (< 1%), and disease progression (< 1%). In the safety population of the GEM studies, a higher percentage of VTD patients received fewer cycles of therapy compared with VRD. For example, 4.4% *vs* 6.2% of patients received three or fewer cycles of VRD *vs* VTD. This trend continued, with 6.1% *vs* 10.8% of patients receiving four or fewer cycles, and 7.0% *vs* 13.8% of patients receiving five or fewer cycles of VRD *vs* VTD, respectively. Thus, more patients receiving VRD *vs* VTD initiated the protocol-defined sixth cycle of induction.

In the IFM studies, 33% and 18% of patients had at least one TEAE leading to dose reduction with VRD *vs* VTD, most commonly peripheral neuropathy (26% *vs* 14%, respectively). At least one TEAE leading to treatment discontinuation occurred in 6% and 11% of patients treated with VRD and VTD. The most common TEAEs leading to discontinuation were peripheral neuropathy (3%) with VRD and peripheral neuropathy (7%) and pulmonary embolism (2%) with VTD. The percentage of patients in the safety population of the IFM studies who received three or fewer cycles was 5.3% for both VRD and VTD.

## 4 Discussion

In the absence of a prospective RCT comparing VRD and VTD, the established methodology of an integrated analysis was used to compare these regimens in TE NDMM. Using patient-level data from the GEM and IFM studies, the analysis met its primary endpoint and demonstrated the noninferiority of VRD *vs* VTD. Furthermore, VRD induction showed superiority by achieving a statistically significant and clinically relevant improvement in ≥ VGPR rate over VTD when six treatment cycles were compared in the GEM studies (66.3% *vs* 51.2%; *P* = .00281). Additionally, the improvement in ≥ VGPR rate from post induction to post ASCT was more notable with VRD than with VTD (rising to 74.4% *vs* 53.5%). Increasing ≥ VGPR rates over the course of treatment, undetectable MRD, and 2-year PFS rates further supported the benefit of VRD over VTD. The difference in ≥ VGPR rates was more notable in the GEM studies, which featured longer cycles than the IFM studies. It is also important to note that the differences in cycle length and overall treatment duration in the GEM studies may have contributed to a greater ≥ VGPR rate than what might be expected in the clinic. These considerations further highlight the importance of cycle length and treatment duration for achieving a clinically meaningful response during induction. While length of induction therapy in GEM2005 and GEM2012 (6 cycles/24 weeks) was longer than that of some other recent phase 3 trials, such as CASSIOPEIA (34), studies incorporating 6 cycles of induction with VTD have produced similar improvements in CR rates from pre- to posttransplant compared with those using 3 cycles of induction with VTD (18, 19). Further, differences in the length of induction therapy among these trials may reflect variations in regional practices. The current ESMO guidelines recommend 4 to 6 cycles of induction with or without consolidation (4).

Although safety results from the GEM2005 study were not included in this analysis due to limited access, safety data reported in the GEM2005 primary manuscript and safety data reported here for GEM2012 and the IFM studies showed that TEAEs were generally consistent with the known safety profiles of lenalidomide, bortezomib, thalidomide, and dexamethasone. Differences in the most common TEAEs between the regimens were largely consistent with the toxicities of lenalidomide and thalidomide. Overall, TEAEs with the VRD regimen were manageable, and the tolerability profile compared well with that of VTD, with fewer TEAEs leading to discontinuation. Peripheral neuropathy due to thalidomide often increases in frequency with long-term use (16, 17, 35, 36). In addition to the effects of lenalidomide *vs* thalidomide in the VRD and VTD regimens (including the dose of thalidomide used), TEAEs should be considered in the context of the different bortezomib routes and frequencies of administration used in these studies. Bortezomib was given subcutaneously in GEM2012 and IFM 2013-04 and intravenously in GEM2005 and IFM 2009. Additionally, bortezomib dose intensity was higher when administered in 3-week cycles in the IFM studies *vs* 4-week cycles in the GEM studies. The efforts made to improve the tolerability of induction regimens were important, as they may have allowed delivery of the full number of planned cycles. This could increase depth of response and lead to improved survival outcomes. One can hypothesize that a weekly subcutaneous bortezomib schedule could further improve tolerability compared with a twice-weekly schedule.

The survival data (PFS and OS) should be interpreted with caution considering the key study design differences. While GEM VRD and VTD trials had largely parallel designs (with six cycles of induction followed by ASCT), post-ASCT treatment differed between the trials. For example, patients in the VRD trial received VRD consolidation (and could enroll in a maintenance study of lenalidomide + dexamethasone ± ixazomib [NCT02406144]), and those in the VTD trial could receive post-ASCT maintenance with thalidomide, thalidomide + bortezomib, or interferon-α2b. Additionally, follow-up times differed between the trials (median 24.0 *vs* 48.4 months for VRD *vs* VTD). To address this, we used the event-free rate at 2 years, which mitigates the differences in median follow-up and number of events and is considered a more representative comparison between the two cohorts. The study designs were more divergent in the IFM trials. Arm A of IFM 2009, which was used for the VRD data analyzed, did not include ASCT and instead included a total of eight cycles of VRD. Due to differences in duration of induction between the IFM studies, we chose to include Arm A from the IFM 2009 study (eight 3-week cycles of VRD [24 weeks]) to permit comparison with the IFM 2013-04 study (four 3-week cycles of VTD [12 weeks]). The VTD study used ASCT following four cycles of VTD induction. Follow-up times also differed between the trials (median 35.0 *vs* 16.6 months for VRD *vs* VTD). All these factors may have affected the reported survival outcomes.

Another point to consider is that different MRD sensitivity thresholds were used across these studies. In GEM2005, 10^−4^ was used since the technology for 10^−6^ was not available at the time the study was conducted. Compared with MRD 10^−5^ and 10^−6^, MRD 10^−4^ is a threshold at which more MRD-positive disease may go undetected, and MRD positivity has been associated with less-durable responses (37). While current response criteria have established an MRD threshold of 10^−5^, evidence has emerged that 10^−6^ may be more clinically relevant. Further, achievement of MRD negativity at 10^−6^ has been associated with longer PFS compared with 10^−5^ (37).

This analysis has several limitations, the most notable of which is the cross-trial comparison. Although we used PS-based statistical methods, it is possible that differences in baseline factors among the trials could have confounded the comparison between treatment cohorts. In addition, the analyzed trials had relatively short follow-up durations and are older, having been published between 2012 and 2019 with a database closure of 2017. Since this time, regimens based on the anti-CD38 monoclonal antibodies daratumumab and isatuximab have emerged. However, both VRD and VTD are being used in current practice as backbone therapy in modern quadruplet induction regimens incorporating daratumumab or isatuximab. Further, the comparison of VRD and VTD remains relevant as both of these triplet regimens are recommended in current TE NDMM treatment guidelines, including ESMO, EMN, and ASCO/CCO guidelines. VRD is also designated as a category 1 preferred regimen for primary therapy for TE patients by the National Comprehensive Cancer Network Guidelines^®^, while VTD has a category 1 designation for use in certain circumstances (38).

As with any systematic analysis, the applicability of these results to the real-word setting is of interest, and different practice patterns in the Europe and the US should be acknowledged. In Europe, VTD is a standard of care for patients with TE NDMM (34), whereas in the US, VRD is considered the optimal induction regimen for these patients (39). Therefore, the results of this analysis will have different implications for each respective setting. The inclusion of younger, healthier patients (age <65 years with ECOG performance status of ≤ 3) may not be generally reflective of a real-world patient population. Lastly, the percentage of patients with undetermined cytogenetic risk included in each trial was unbalanced, ranging from 8% to 36%; therefore, it is likely that an analysis of real-world patients may have a more equitable distribution of risk groups.

Although some studies did not meet the criteria for inclusion in the integrated analysis, their results further support the use of VRD. For example, the DSMM XIV, GMMG-HD6, and SWOG S0777 studies showed VRD to be active and well tolerated (26, 27, 40), with the latter supporting the recent European approval of VRD for transplant-ineligible patients. In Myeloma XI, responses to induction were deeper with cyclophosphamide + lenalidomide + dexamethasone *vs* cyclophosphamide + thalidomide + dexamethasone (≥ VGPR rates, 60.4% *vs* 52.9%, respectively) (41). Responses also deepened with additional cycles, highlighting the importance of a tolerable regimen to maximize the number of cycles that can be given (42). Of note, although induction was stopped due to toxicity at a similar rate for cyclophosphamide + lenalidomide + dexamethasone *vs* cyclophosphamide + thalidomide + dexamethasone (5.0% *vs* 6.7%, respectively), dose modifications of lenalidomide were less frequent than of thalidomide (38.3% *vs* 73.6%, respectively) (41). Together, these results supported the advantage of lenalidomide- *vs* thalidomide-containing regimens. Moreover, results of several other studies suggest that there is a role for VRD as consolidation therapy in NDMM (25, 43). For example, in the EMN02/HOVON95 trial, consolidation therapy with VRD followed by maintenance with lenalidomide improved PFS in patients with NDMM *vs* maintenance alone (43). However, the benefit observed in this trial is possibly due to use of a suboptimal induction regimen—some other trials have not demonstrated the same effect (44).

Given the incurable nature of MM and the fact that patients with MM will ultimately experience relapse, selecting the ideal induction regimen is critical for minimizing disease burden and promoting durable survival outcomes. In lieu of a direct comparison between VRD and VTD, it is our hope that this analysis can be used to help inform these treatment decisions and address clinical questions that are of particular importance to clinicians who treat patients with NDMM. Overall, the results of this integrated analysis provide evidence demonstrating the benefit of VRD over VTD as induction treatment in TE patients with NDMM. These results support the inclusion of VRD as a preferred regimen for primary therapy for transplant candidates (4–6).

## Data availability statement

The original contributions presented in the study are included in the article/**Supplementary Material**, further inquiries can be directed to this link: https://www.bms.com/researchers-and-partners/independent-research/data-sharing-request-process.html.

## Author contributions

All authors contributed to the concept and design of the work, acquisition, analysis, or interpretation of data for the work; contributed to the drafting of the work; revised the manuscript critically for important intellectual content; approved the final version to be published; and agree to be accountable for all aspects of the work.
