# Supplementary material for: Integrated analysis of randomized controlled trials evaluating bortezomib + lenalidomide + dexamethasone or bortezomib + thalidomide + dexamethasone induction in transplant-eligible newly diagnosed multiple myeloma
Source: Front Oncol. 2023 Nov 2;13:1197340. doi: 10.3389/fonc.2023.1197340 (PMC10652744; doi:10.3389/fonc.2023.1197340)
Supplement: Supplementary file 1 [file DataSheet_1.docx]

**Supplemental Information**

*Search details*

Searches were performed using ClinicalTrials.gov, ClinicalTrialsRegister.eu, PubMed, Medline, Embase, and Biosis. Search terms included phase 3, phase III, lenalidomide, Revlimid, thalidomide, Thalomid, bortezomib, Velcade, RVD, VRD, BLD, BRD, BTD, TBD, VTD, TVD, and myeloma. A database of studies supported by Celgene, a Bristol-Myers Squibb Company, was also reviewed.

*Description of balance diagnostics in the PS model*

Balance diagnostics were performed with the VRD and VTD cohorts to confirm that the PS model was adequately specified. The following were compared between the VRD and VTD cohorts:

- Distribution of PS based on a histogram plot
- Means of continuous and frequency (percentages) of dichotomous baseline covariates
- Variance of continuous variables
- Standardized difference (to quantify differences in means or frequency [percentages])
- Difference in mean covariate values across strata using a regression model with covariate as the dependent variable

*Statistical hypothesis*

The Cochran-Mantel-Haenszel test stratified on the stratum based on the quintiles of the PS was used to estimate the difference in ≥ VGPR rates and 95% CI of achieving ≥ VGPR with VRD vs VTD. The same method was used to estimate differences in undetectable MRD rates and 95% CI in the PETHEMA GEM studies. The test hypothesis was that the postinduction ≥ VGPR rate of VRD was at least that of VTD – δ (the margin), ie, H_0_: p(VRD) – p(VTD) ≤ –δ vs H_1_: p(VRD) – p(VTD) > –δ. The noninferiority margin (11.3%) was selected using historical data; a margin of 10% did not represent a substantial difference in treatment effect and was within normal variance between two treatment regimens in similar patient populations. Following a positive finding of noninferiority, superiority could be assessed as to whether the 95% CI lay entirely above 0.

*Peripheral neuropathy*

Peripheral neuropathy was summarized using a group term, which included the terms neuropathy peripheral, peripheral sensory neuropathy, polyneuropathy, burning sensation, hypoesthesia, paresthesia, autonomic neuropathy, peripheral motor neuropathy, dysesthesia, neurotoxicity, hyperesthesia, discomfort, asthenia, peripheral sensorimotor neuropathy, neuralgia, gait disturbance, hyporeflexia, hypotonia, formication, peroneal nerve palsy, sensory loss, muscle atrophy, muscular weakness, and abnormal coordination.

Supplemental Figure 1. Forest plot of postinduction ≥ VGPR rate in the GEM studies.


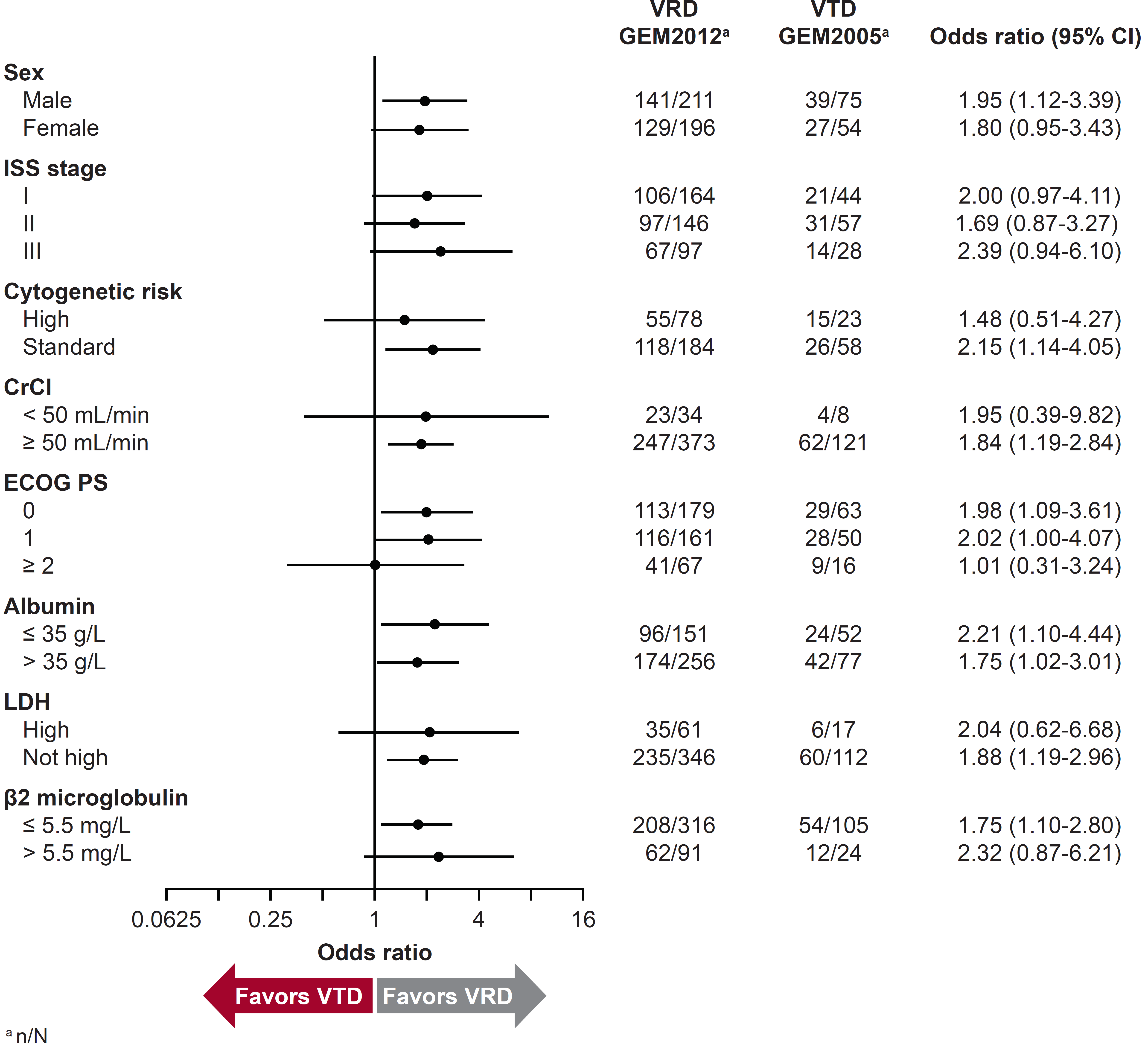


CrCl, creatinine clearance; ECOG PS, Eastern Cooperative Oncology Group performance status; ISS, International Staging System; LDH, lactate dehydrogenase; n/N, number of patients with ≥ VGPR/number of patients; VGPR, very good partial response; VRD, bortezomib, lenalidomide, and dexamethasone; VTD, bortezomib, thalidomide, and dexamethasone.

Supplemental Figure 2. Forest plot of postinduction ≥ VGPR rate in the IFM studies.

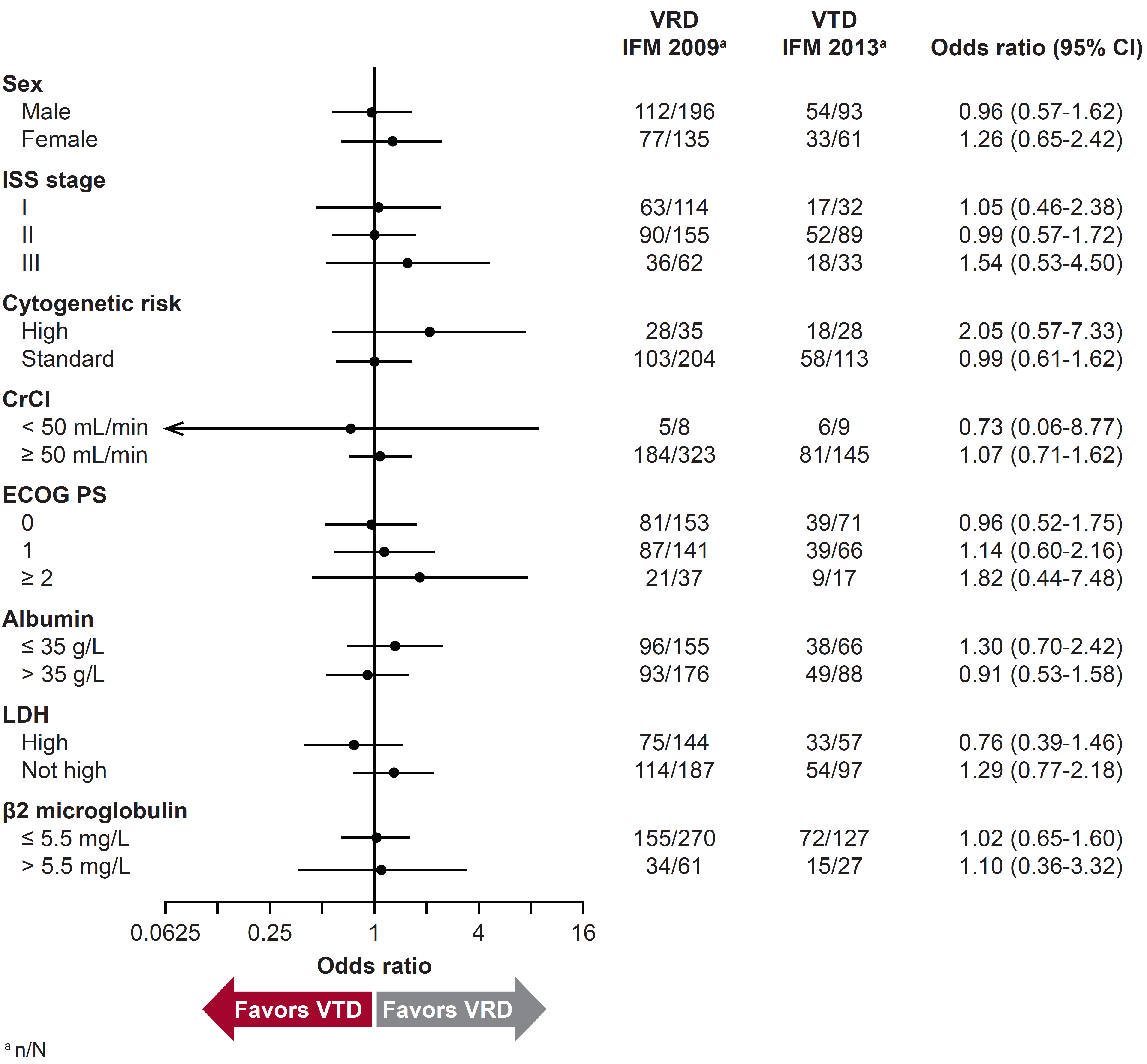
 CrCl, creatinine clearance; ECOG PS, Eastern Cooperative Oncology Group performance status; ISS, International Staging System; LDH, lactate dehydrogenase; n/N, number of patients with ≥ VGPR/number of patients; VGPR, very good partial response; VRD, bortezomib, lenalidomide, and dexamethasone; VTD, bortezomib, thalidomide, and dexamethasone.

Supplemental Table 1. Summary of the phase 3 studies considered for the integrated analysis as of the finalization of the integrated analysis statistical analysis plan (13 November 2017).

| **Trial name**  **(NCT number)** | **Study Design** | **Treatment Regimen** | **Patients, n** | | **Study Dates** | **Included** | | **Reason for Exclusion** |
| --- | --- | --- | --- | --- | --- | --- | --- | --- |
| *VRD studies* |  | | | | | | | |
| PETHEMA GEM2012MENOS65 (NCT01916252)(24) | Phase 3, randomized, controlled, open-label, multicenter study  18-65 years, TE NDMM  Spain | **Induction**: VRD (4-week cycles)  **Conditioning**: MEL200 (Arm A) vs Bu-Mel (Arm B)  **ASCT**  **Consolidation:** VRD | 458 randomized, 460 planned | | Sept 2013 to Nov 2015 | Yes | | NA |
| IFM 2009 (NCT01191060)(25) | Phase 3, randomized, controlled, open-label, multicenter study  18-65 years, TE NDMM  France, Belgium, and Switzerland | **Induction**: VRD (arms A and B; 3-week cycles; randomization after first cycle)  **MEL200 + ASCT** (arm B only)  **Consolidation**: VRD (arms A and B)  **Maintenance**: LEN (arms A and B) | 700 randomized, 700 planned | | Nov 2010 to Nov 2012 | Yes | | NA |
| DFCI 2009 (NCT01208662) | Phase 3, randomized, controlled, open-label, multicenter study  ≤ 65 years, TE NDMM  USA | **Induction**: VRD (arms A and B; 3‑week cycles; randomization after first cycle)  **ASCT** (arm B only)  **Consolidation**: VRD (arms A and B)  **Maintenance**:  LEN (arms A and B) | 368 randomized, 660 planned | | FPI: Oct 2010 | No | | Primary endpoint not met |
| DSMM XIV  (NCT01685814)(27) | Phase 3, randomized, controlled, open-label, multicenter study; double 2 × 2 factorial design  18-65 years, TE NDMM  Germany | **Induction:** VRD (3-week cycles) vs RAD (4-week cycles)  **ASCT**  **Consolidation/maintenance**:  (1) ≥ VGPR: 2nd ASCT + LEN maintenance vs LEN maintenance  (2) < VGPR: 2nd ASCT + LEN maintenance vs alloSCT + low‑dose LEN maintenance | 476 randomized, 406 planned | | May 2012 to Jun 2016 | No | | Lack of an agreement for access to patient-level data |
| ECOG E1A11; ENDURANCE (NCT01863550) | Phase 3, randomized, controlled, open-label, multicenter study  ≥ 18 years, TE or TNE NDMM  USA | **Induction:** VRD (3-week cycles) vs CRd (4‑week cycles)  **Maintenance (randomized)**: LEN (2 years vs until PD or unacceptable toxicity) | 223 randomized, 1080 planned | | FPI: Dec 2013 | No | | Did not include ASCT in first line |
| EFC12522; IMROZ (NCT03319667)(37) | Phase 3, randomized, controlled, open-label, crossover, multicenter study  18-80 years, TNE NDMM  Multinational (not yet specified) | **Induction:** Arm 1: Isa + VRD (6-week cycles)  Arm 2: VRD (6-week cycles)  **Maintenance (until PD):** Isa + VRD (arm 1), VRD (arm 2)  **Crossover:** Isa + Rd (when applicable) | 440 planned | | FPI: Dec 2017 | No | | Enrollment ongoing (primary endpoint not met) |
| GMMG-HD6 (NCT02495922)(38) | Phase 3, randomized, controlled, open-label, multicenter study  18-70 years, TE NDMM  Germany | **Induction:** VRD (arms A1 and A2) vs VRD‑Elo (arms B1 and B2) (3‑week cycles)  **ASCT**  **Consolidation:** VRD (arms A1 and B1) vs VRD-Elo (arms A2 and B2)  **Maintenance:** Rd (arms A1 and B1) vs Rd‑Elo (arms A2 and B2) | 564 randomized, 516 planned | | FPI: Jun 2015 | No | | Primary endpoint not met |
| SWOG S0777 (NCT00644228)(26) | Phase 3, randomized, controlled, open-label, multicenter study  ≥ 18 years, TE or TNE NDMM  USA and Saudi Arabia | **Initial therapy**: VRD (3‑week cycles) vs Rd (4‑week cycles)  **Continued therapy:** Rd until disease relapse | 523 randomized, 440 planned | | Jul 2008 to Feb 2012 | No | | Did not include ASCT in first line |
| Identifier NS(39) | Phase 3, randomized, prospective study  Age criterion NS (range, 31-70 years), TE or TNE NDMM  India | **Induction (randomized):** VRD vs Rd (4-week cycles) | 144 randomized (number planned NS) | | Sep 2014 to Oct 2016 | No | | Did not include ASCT in first line |
| *VTD studies* |  | | | | | | | |
| PETHEMA GEM05MENOS65 (NCT00461747)(18) | Phase 3, randomized, controlled, open-label, multicenter study with double randomization  ≤ 65 years, TE NDMM  Spain | **Induction:** VBMCP/VBAD (alternating 5-/4‑week cycles) followed by BORT (3‑week cycles) (arm A) vs  TD (4-week cycles) (arm B) vs  VTD (4-week cycles) (arm C)  **MEL200 + ASCT**  **Maintenance:** IFN-a 2b (group M1), THAL (group M2), or VT (group M3) | 390 randomized, 390 planned | | Apr 2006 to Aug 2009 | Yes | | NA |
| IFM 2013-04 (NCT01971658)(16) | Phase 3, randomized, controlled, open-label, multicenter study; ASCT outside of the protocol  18-65 years, TE NDMM  France | **Induction:** VTD vs VCD (3‑week cycles)  **ASCT** | 340 randomized, 358 planned | | Nov 2013 to Mar 2015 | Yes | | NA |
| GIMEMA MMY‑3006 (NCT01134484)(19) | Phase 3, randomized, controlled, open-label, multicenter study  18-65 years, TE NDMM  Italy | **Induction**: VTD vs TD (3‑week cycles)  **ASCT × 2**  **Consolidation:** VTD vs TD (2 cycles) | 480 randomized (474 ITT), 450 planned | | May 2006 to Apr 2008 | No | | Lack of an agreement for access to patient-level data |
| IFM 2007-02 (NCT00910897)(40) | Phase 3, randomized controlled, open-label, multicenter study; reduced dose of BORT in VTD arm  < 65 years, TE NDMM  Belgium and France | **Induction:** vtD vs VD (3-week cycles)  **ASCT** | | 199 randomized, 205 planned | Mar 2008 to Jan 2009 | No | Reduced dose of BORT in the VTD arm | |
| IFM 2015-01; CASSIOPEIA (NCT02541383) | Phase 3, randomized, controlled, open-label, parallel, multicenter study  18-65 years, TE NDMM  Belgium, France, Luxembourg, the Netherlands | **Induction:** VTD (arm A) vs VTD + Dara (arm B) (4-week cycles)  **ASCT**  **Consolidation:** VTD (arm A) or VTD + Dara (arm B)  **Maintenance:** Dara (arm B only) | 1080 planned | | FPI: Sept 2015 | No | | Enrollment ongoing (primary endpoint not met) |
| Total Therapy 4b (NCT00734877)(41,42) | Phase 3, randomized, controlled, open-label, parallel study  ≥ 18 and < 75 years, low-risk NDMM  USA | **Induction:** VTDPACE (cycle length NS)  **MEL-based tandem transplant**  **Consolidation:** VTDPACE (dose reduced)  **Maintenance:** VRD | 289 randomized, 400 planned | | FPI: Jul 2008 | No | | Enrollment ongoing (primary endpoint not met) |
| US Community-Based Trial (NCT00507416)(20) | Phase 3b, randomized, open-label, multicenter, community-based trial  ≥ 65 years, TNE NDMM  USA | **Induction:** VTD vs VD vs VMP (3‑week cycles)  **Maintenance:** BORT | 502 randomized, 502 planned | | Jun 2007 to Mar 2010 | No | | Study population not eligible for transplant |

alloSCT, allogenic stem cell transplant; ASCT, autologous stem cell transplant; BORT, bortezomib; Bu-Mel, busulfan + melphalan; CRd, carfilzomib + lenalidomide + dexamethasone; Dara, daratumumab; FPI, first patient included; IFN, interferon; Isa, isatuximab; ITT, intent-to-treat; LEN, lenalidomide; MEL200, melphalan 200 mg/m^2^; NA, not applicable; NDMM, newly diagnosed multiple myeloma; NS, not specified; PD, progressive disease; RAD, lenalidomide + doxorubicin + dexamethasone; Rd, lenalidomide + dexamethasone; Rd-Elo, lenalidomide + dexamethasone + elotuzumab; TD, thalidomide + dexamethasone; TE, transplant eligible; THAL, thalidomide; TNE, transplant noneligible; VBAD, vincristine + carmustine + doxorubicin + dexamethasone; VBMCP, vincristine + carmustine + melphalan + cyclophosphamide + prednisone; VCD, bortezomib + cyclophosphamide + dexamethasone; VD, bortezomib + dexamethasone; VGPR, very good partial response; VMP, bortezomib + melphalan + prednisone; VRD, bortezomib + lenalidomide + dexamethasone; VRD-Elo, bortezomib + lenalidomide + dexamethasone + elotuzumab; vtD, reduced dose of bortezomib and thalidomide + dexamethasone; VTD, bortezomib + thalidomide + dexamethasone; VTDPACE, bortezomib + high‑dose dexamethasone + thalidomide + cisplatin + doxorubicin + cyclophosphamide + etoposide.

Supplemental Table 2. Summary of overall propensity score.

|  | **VRD**  **GEM2012**  **N = 407** | **VTD**  **GEM2005**  **N = 129** | **VRD**  **IFM 2009**  **N = 331** | **VTD**  **IFM 2013-04**  **N = 154** |
| --- | --- | --- | --- | --- |
| Mean | 0.77 | 0.73 | 0.82 | 0.78 |
| Standard deviation | 0.083 | 0.088 | 0.075 | 0.089 |
| Minimum, maximum | 0.51, 0.95 | 0.49, 0.95 | 0.54, 0.96 | 0.46, 0.94 |
| Median | 0.78 | 0.74 | 0.84 | 0.77 |
| 25%, 75% | 0.71, 0.83 | 0.68, 0.78 | 0.78, 0.87 | 0.73, 0.85 |

25%, 25th percentile; 75%, 75th percentile; VRD, bortezomib, lenalidomide, and dexamethasone; VTD, bortezomib, thalidomide, and dexamethasone.

Supplemental Table 3. Baseline patient and disease characteristics in PS-stratified cohorts and ITT populations.

|  | **PS-Stratified**  **VRD**  **GEM2012**  **n = 407** | **ITT**  **VRD**  **GEM2012**  **n = 458** | **PS-Stratified**  **VTD**  **GEM2005**  **n = 129** | **ITT**  **VTD**  **GEM2005**  **n = 130** | **PS-Stratified**  **VRD**  **IFM 2009**  **n = 331** | **ITT**  **VRD**  **IFM 2009**  **n = 350** | **PS-Stratified**  **VTD**  **IFM 2013-04**  **n = 154** | **ITT**  **VTD**  **IFM 2013-04**  **n = 169** |
| --- | --- | --- | --- | --- | --- | --- | --- | --- |
| Median age, years | 57 | 58 | 57 | 57 | 59 | 58 | 59 | 59 |
| Range | 31-65 | 31-65 | 33-65 | 33-65 | 28-65 | 28-65 | 34-65 | 34-65 |
| Male, n (%) | 211 (51.8) | 240 (52.4) | 75 (58.1) | 76 (58.5) | 196 (59.2) | 208 (59.4) | 93 (60.4) | 103 (60.9) |
| ECOG PS, n (%) |  |  |  |  |  |  |  |  |
| 0 | 179 (44.0) | 195 (42.6) | 63 (48.8) | 64 (49.2) | 153 (46.2) | 165 (47.1) | 71 (46.1) | 80 (47.3) |
| 1 | 161 (39.6) | 182 (39.7) | 50 (38.8) | 50 (38.5) | 141 (42.6) | 148 (42.3) | 66 (42.9) | 70 (41.4) |
| ≥ 2 | 67 (16.5) | 78 (17.0) | 16 (12.4) | 16 (12.3) | 37 (11.2) | 37 (10.6) | 17 (11.0) | 17 (10.1) |
| Missing | 0 | 3 (0.7) | 0 | 0 | 0 | 0 | 0 | 2 (1.2) |
| ISS stage, n (%) |  |  |  |  |  |  |  |  |
| I | 164 (40.3) | 179 (39.1) | 44 (34.1) | 44 (33.8) | 114 (34.4) | 122 (34.9) | 32 (20.8) | 38 (22.5) |
| II | 146 (35.9) | 166 (36.2) | 57 (44.2) | 58 (44.6) | 155 (46.8) | 162 (46.3) | 89 (57.8) | 94 (55.6) |
| III | 97 (23.8) | 107 (23.4) | 28 (21.7) | 28 (21.5) | 62 (18.7) | 66 (18.9) | 33 (21.4) | 37 (21.9) |
| Missing | 0 | 6 (1.3) | 0 | 0 | 0 | 0 | 0 | 0 |
| LDH, n (%) |  |  |  |  |  |  |  |  |
| Elevated | 61 (15.0) | 65 (14.2) | 17 (13.2) | 17 (13.1) | 144 (43.5) | 144 (41.1) | 57 (37.0) | 59 (34.9) |
| Not elevated | 346 (85.0) | 376 (82.1) | 112 (86.8) | 113 (86.9) | 187 (56.5) | 187 (53.4) | 97 (63.0) | 99 (58.6) |
| Missing | 0 | 17 (3.7) | 0 | 0 | 0 | 19 (5.4) | 0 | 11 (6.5) |
| CrCl group, n (%) |  |  |  |  |  |  |  |  |
| < 50 mL/min | 34 (8.4) | 36 (7.9) | 8 (6.2) | 8 (6.2) | 8 (2.4) | 8 (2.3) | 9 (5.8) | 10 (5.9) |
| ≥ 50 mL/min | 373 (91.6) | 404 (88.2) | 121 (93.8) | 122 (93.8) | 323 (97.6) | 342 (97.7) | 145 (94.2) | 159 (94.1) |
| Missing | 0 | 18 (3.9) | 0 | 0 | 0 | 0 | 0 | 0 |

CrCl, creatinine clearance; ECOG PS, Eastern Cooperative Oncology Group performance status; ISS, International Staging System; ITT, intent-to-treat; LDH, lactate dehydrogenase; PS, propensity score; VRD, bortezomib, lenalidomide, and dexamethasone; VTD, bortezomib, thalidomide, and dexamethasone.
